# Association between preoperative platelet count and transjugular intrahepatic portosystemic shunt procedure-related hemorrhage in cirrhotic patients: a retrospective study

**DOI:** 10.3389/fmed.2025.1683046

**Published:** 2025-11-18

**Authors:** Ying Li, Jisen Xu, Xin Quan, Bo Wei, Yang Tai, Hao Wu

**Affiliations:** Department of Gastroenterology and Hepatology, West China Hospital, Sichuan University, Chengdu, China

**Keywords:** cirrhosis, transjugular intrahepatic portosystemic shunt-procedure-related hemorrhage, thrombocytopenia, platelet count, portal hypertension

## Abstract

**Objective:**

Transjugular intrahepatic portosystemic shunt (TIPS) is a high-bleeding-risk vascular intervention. The association between TIPS-related hemorrhage and preprocedural platelet (PLT) count remains unclear.

**Methods:**

This was a retrospective cohort study including patients receiving TIPS procedures for complications related to portal hypertension due to liver cirrhosis between 2011 and 2022. Logistic regression and subgroup analyses were performed to evaluate the relationship between TIPS-related hemorrhage and preprocedural PLT count. Patients were divided into two groups based on a PLT threshold of 50 × 10^9^/L. Furthermore, patients with PLT count < 50 × 10^9^/L were stratified into two subgroups based on a threshold of 20 × 10^9^/L. The primary endpoint was TIPS procedure-related hemorrhage.

**Results:**

A total of 632 patients with liver cirrhosis who underwent TIPS were categorized into two groups based on their PLT counts of 50 × 10^9^/L. The incidence of TIPS-related hemorrhage was 2.8% in the PLT < 50 × 10^9^/L group (*n* = 417) and 1.9% in the PLT ≥ 50 × 10^9^/L group (*n* = 215) (95% CI: 0.23–1.97; *P* = 0.480). In subgroup analysis, the bleeding rate was 0% in PLT < 20 × 10^9^/L group (*n* = 10) and 2.9% in PLT 20–50 × 10^9^/L group (*n* = 205). No statistically significant intergroup differences in bleeding rates were found (95% CI: 0–9.7; *P* = 0.501). In the univariate and multivariate analysis, advanced age is the independent risk factor for TIPS-related bleeding (OR = 1.054, 95% CI: 1.006–1.105, *P* = 0.028).

**Conclusion:**

This study revealed that preoperative PLT count is not associated with TIPS procedure-related hemorrhage in patients with cirrhosis. Patient age should be carefully considered in the preoperative assessment.

## 1 Introduction

Transjugular intrahepatic portosystemic shunt (TIPS) is an effective intervention for reducing portal hypertension and is utilized in managing complications related to portal hypertension in patients with cirrhosis, including refractory ascites and variceal rebleeding (1). However, the American Association for the Study of Liver Diseases (AASLD) classifies TIPS as a high bleeding risk procedure for patients with cirrhosis (2). TIPS procedure-related hemorrhages, primarily consisting of intraperitoneal bleeding and hemobilia, are observed in about 1%–5% of patients (3). This complication is life-threatening and is linked to an elevated mortality rate (4, 5).

Thrombocytopenia, a contributor to bleeding, is observed in up to 78% of cirrhotic patients (6, 7). Given the lack of prospective data to establish definitive platelet (PLT) thresholds for safe TIPS placement, current guideline recommendations are not harmonized. North American practice-based guidelines for TIPS in portal hypertension do not specify a target PLT threshold for cirrhotic patients (8). TIPS Stent-Shunt in the Management of Portal Hypertension indicates that patients with PLT < 75 × 10^9^/L may not benefit from TIPS for ascites (4). In contrast, Chinese guidelines consider PLT < 20 × 10^9^/L is a relative contraindication for TIPS placement (9). PLT transfusions prior to invasive procedures to correct thrombocytopenia and reduce the risk of procedure-related bleeding are commonly performed, particularly in patients with a PLT count < 50 × 10^9^/L (10). Nonetheless, considering the complex rebalancing of coagulation in cirrhosis patients and the potential risks associated with PLT transfusions, there remains debate over the necessity of preoperative PLT transfusions for patients with PLT counts below 50 × 10^9^/L (11).

Bleeding disorders have historically posed a significant clinical challenge. However, inappropriate clotting has recently emerged as a major concern due to alterations in the hemostatic equilibrium (12, 13). In a study conducted by Chen et al. (14) on cirrhotic patients with thrombocytopenia, it was determined that the PLT count was not correlated with bleeding events post-TIPS and did not serve as a predictor for bleeding. Similarly, Shah et al. (15) reported that neither the PLT nor the international normalized ratio (INR) could predict bleeding during invasive procedures in patients with cirrhosis. However, a study of 50 liver transplant candidates with PLT counts below 125 × 10^9^/L revealed that 20% of patients experienced bleeding complications following procedures, all of whom had PLT counts below 75 × 10^9^/L (16). The conflicting data in the aforementioned literature likely stems from the broad inclusion criteria, which encompass cirrhotic patients undergoing various invasive procedures with differing bleeding risks. To date, no dedicated study has specifically examined the relationship between preoperative PLT counts and hemorrhagic complications in TIPS procedures, known to carry a high bleeding risk. It remains unclear whether this complication is associated with thrombocytopenia, and no PLT threshold has been recommended for safe TIPS creation (8). Clinicians often face complex decisions balancing the heightened procedural bleeding risk due to thrombocytopenia against the urgent need for TIPS placement.

Therefore, we conducted a retrospective study to evaluate whether the occurrence of TIPS procedure-related hemorrhage in cirrhotic patients correlates with preoperative PLT counts and to pinpoint the risk factors for hemorrhage linked to TIPS procedures.

## 2 Materials and methods

### 2.1 Patients

We conducted a retrospective analysis of consecutive decompensated cirrhotic patients admitted to West China Hospital who underwent TIPS from July 2011 to February 2022. The study protocol was approved by the Ethics Committee of West China Hospital (protocol number: 104). Informed consent was waived because the data have been anonymized. The inclusion criteria were as follows: (1) age > 18 years; (2) diagnosis of decompensated cirrhosis based on liver biopsy or standard clinical, biochemical, and imaging parameters; and (3) successful TIPS placement. Patients were excluded if they met any of the following criteria: (1) missing necessary clinical data; (2) hepatocellular carcinoma exceeding the Milan criteria or other extrahepatic malignancies; (3) previous TIPS placement; (4) administration of anticoagulants, antiplatelet medications, platelet-enhancing agents within 2 weeks prior to TIPS; (5) preoperative PLT transfusion; (6) hematological and immunological diseases; (7) liver cirrhosis with portal cavernous transformation. Patients were divided into two groups based on a PLT threshold of 50 × 10^9^/L: those with PLT counts < 50 × 10^9^/L and those with PLT counts ≥ 50 × 10^9^/L. Furthermore, patients with PLT counts < 50 × 10^9^/L were stratified into two subgroups based on a threshold of 20 × 10^9^/L: those with PLT counts < 20 × 10^9^/L and those with PLT counts between 20 and 50 × 10^9^/L.

### 2.2 TIPS procedure

The TIPS procedure was performed by a dedicated team of well-trained practitioners. All patients received 8–10 mm stents (Bare-metal stent; or Viatorr stent). Varices with diameters greater than 5 mm were embolized with coils (MReye; Cook, Bloomington, IN, US). The criteria for TIPS technical success included stent patency on post-procedural DSA and complete disappearance of variceal blood flow.

### 2.3 Clinical data

All clinical, biochemical, and radiological data were obtained from the electronic medical records at the time point nearest to the TIPS procedure. The patient’s past medical history, including diabetes mellitus, chronic kidney disease (CKD) and hypertension, was also documented. Ascites is defined as the pathological accumulation of at least 200 mL of fluid in the peritoneal cavity. It is graded from 1 to 3 based on the sonographic depth of the fluid: Grade 1 (<3 cm), Grade 2 (3–10 cm), and Grade 3 (>10 cm) (17). The primary endpoint was TIPS procedure-related hemorrhage, defined as bleeding that occurred during the operation or within the first week following the TIPS procedure, meeting at least one of the following criteria (18, 19): (1) hemorrhagic ascites on paracentesis with hemodynamic instability (systolic BP < 90 mmHg, tachycardia > 120 bpm) or a hemoglobin drop ≥ 2 g/dL; (2) confirmation by abdominal CT or ultrasound; and (3) angiography of vessels showing contrast agent extravasation.

### 2.4 Statistical analysis

Continuous variables were expressed as medians (interquartile ranges, IQRs) and compared using *t*-tests or Mann–Whitney U tests. Categorical variables were expressed as frequencies (percentages) and compared using the chi-square test or Fisher’s exact test. Logistic regression was employed to examine variables associated with TIPS procedure-related hemorrhage. Variables with *P*-values < 0.05 in the univariate analyses were selected for the subsequent multivariate analysis. A *P*-value < 0.05 was considered to indicate statistical significance. All statistical analyses were performed using the statistical package SPSS version 24.0.

## 3 Results

### 3.1 Study population

**FIGURE 1:**
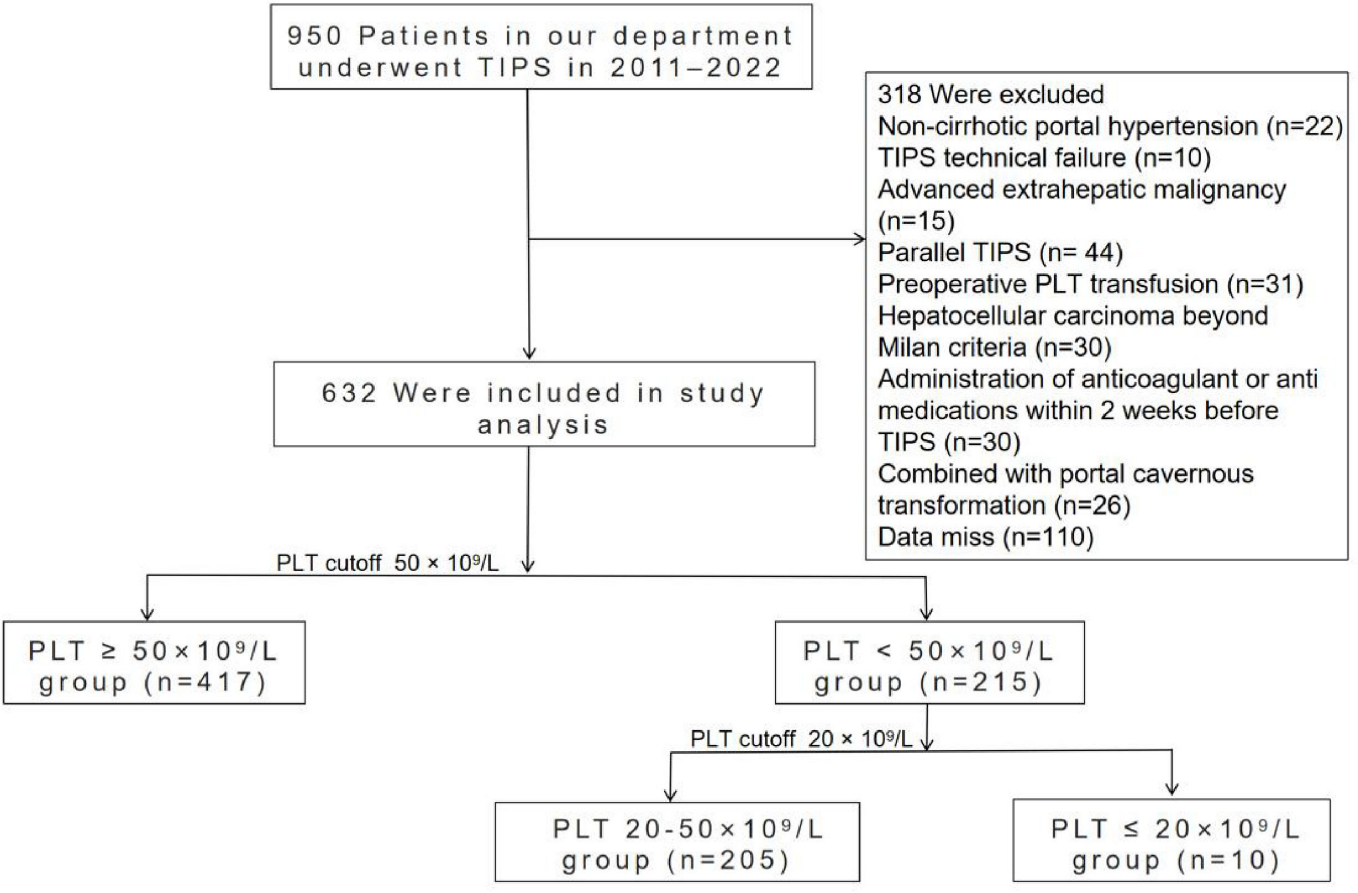
Flow chart showing the study design and participants’ disposition.

During the study period, a total of 950 decompensated cirrhotic patients admitted to West China Hospital who underwent a TIPS procedure were initially considered for the study. Of these, 318 patients were ultimately excluded for the reasons shown in Figure 1, leaving 632 patients eligible for analysis.

The baseline characteristics of patients with PLT counts above or below 50 × 10^9^/L are summarized in Table 1. The main cause of cirrhosis is hepatitis B virus (HBV) infection. The primary indication for TIPS was variceal rebleeding (86.1%). Eighteen patients (2.8%) had a history of CKD, and 102 (16.1%) had diabetes at baseline. The median Model for End-Stage Liver Disease (MELD) score was 10 (IQR, 9–12). Compared with patients with PLT counts ≥ 50 × 10^9^/L, patients with PLT counts < 50 × 10^9^/L had significantly lower fibrinogen (FIB) levels, higher MELD and Child–Pugh scores, INR values, and total bilirubin levels. There was no significant difference in past medical history, indications for TIPS or severity of ascites between the two groups.

**TABLE 1:** Baseline characteristics in group with PLT ≥ 50 × 10^9^/L and group with PLT < 50 × 10^9^/L group.

Characteristics	Total (*n* = 632)	PLT ≥ 50 × 10^9^/L (*n* = 417)	PLT < 50 × 10^9^/L (*n* = 215)	*P*-value
Age (years)	54 (47–65)	58 (49–69)	57 (49–66)	0.333
Male sex, *n* (%)	406 (64.2)	264 (63.3)	142 (66.0)	0.496
Hypertension, *n* (%)	58 (9.2)	45 (10.8)	13 (6.0)	0.050
Chronic kidney disease, *n* (%)	18 (2.8)	14 (3.4)	4 (1.9)	0.284
Splenectomy, *n* (%)	48 (7.6)	47 (11.3)	1 (0.5)	<0.001
Diabetes mellitus, *n* (%)	102 (16.1)	71 (17.0)	31 (14.4)	0.399
Preoperative PPG (mmHg)	19 (16–22)	18 (16–22)	20 (17–25)	0.084
Etiology of liver cirrhosis, *n* (%)				0.061
HBV/HCV	357/17 (59.2)	217/12 (54.9)	140/5 (67.4)	
Alcohol	91 (14.4)	69 (16.5)	22 (10.2)	
PSC/PBC/AIH	0/55/26 (12.8)	0/41/19 (15.4)	0/14/7 (9.8)	
NAFLD	7 (1.1)	6 (1.4)	1 (0.5)	
Others	79 (12.5)	53 (12.7)	26 (12.1)	
Indications for TIPS, *n* (%)				0.448
Recurrent variceal bleeding	544 (86.1)	354 (84.9)	190 (88.4)	
Refractory ascites	79 (12.5)	56 (13.4)	23 (10.7)	
Others	9 (1.4)	7 (1.7)	2 (0.9)	
Ascites, *n* (%)				0.656
Mild	247 (39.1)	157 (37.6)	90 (41.9)	
Moderate	96 (15.2)	62 (14.9)	34 (15.8)	
Severe	63 (10.0)	44 (10.6)	19 (8.8)	
Laboratory				
Hemoglobin (g/L)	82 (71–101)	85 (73–107)	85 (73–103)	0.496
White blood cell (×10^9^/L)	3.05 (2.17–4.42)	3.6 (2.6–5.0)	2.2 (1.8–3.0)	<0.001
Total bilirubin (μmol/L)	19.8 (14.4–28.9)	18.7 (13.7–25.8)	23.1 (16.1–34.8)	<0.001
Aspartate transaminase (U/L)	31 (23–42)	31 (23–41)	32 (24–43)	0.823
Alanine aminotransferase (U/L)	21 (15–30)	21 (14–28)	21 (14–30)	0.802
Albumin (g/L)	33.7 (30.0–38.1)	33.4 (29.4–38.2)	34.2 (30.5–38.4)	0.196
Creatinine (μmol/L)	68 (57–82)	69 (59–86)	70 (60–86)	0.840
Sodium (mmol/L)	139.3 (136.9–141.1)	138.9 (136.3–140.8)	138.9 (136.9–141.1)	0.543
PT (second)	14.2 (13.1–15.5)	13.6 (12.5–14.9)	14.4 (13.6–15.6)	<0.001
INR	1.24 (1.15–1.36)	1.2 (1.1–1.4)	1.3 (1.2–1.4)	<0.001
Fibrinogen (g/L)	1.69 (1.35–2.21)	1.90 (1.53–2.45)	1.56 (1.32–1.99)	<0.001
Child-Pugh score	7 (6–9)	7 (6–8)	8 (6–9)	<0.001
Child-Pugh class, *n* (%)				0.002
A (5–6)	201 (31.8)	149 (35.7)	52 (24.2)	
B (7–9)	358 (56.6)	230 (55.2)	128 (59.5)	
C (10–15)	73 (11.6)	38 (9.1)	35 (16.3)	
MELD score	10 (9–12)	9 (8–12)	11 (9–13)	<0.001

The baseline characteristics of patients in the subgroup with PLT counts < 20 × 10^9^/L (*n* = 10) and those in the group with PLT counts 20–50 × 10^9^/L (*n* = 205) are shown in Table 2. No significant differences in baseline characteristics were observed between the two subgroups.

**TABLE 2:** Baseline characteristics in the group with PLT 20–50 × 10^9^/L and the group with PLT < 20 × 10^9^/L.

Characteristics	PLT 20–50 × 10^9^/L (*n* = 205)	PLT < 20 × 10^9^/L (*n* = 10)	*P*-value
Age (years)	56 (49–66)	58 (52–67)	0.530
Male sex, *n* (%)	136 (66.3)	6 (60.0)	0.679
Hypertension, *n* (%)	12 (5.9)	1 (10.0)	0.591
Chronic kidney disease, *n* (%)	4 (2.0)	0	0.656
Diabetes mellitus, *n* (%)	31 (15.1)	0	0.184
Splenectomy, *n* (%)	1 (0.5)	0	0.825
Preoperative PPG (mmHg)	20 (17–22)	18 (15–21)	0.437
Etiology of liver cirrhosis, *n* (%)			0.866
HBV/HCV	133/5 (67.3)	7/0 (70)	
Alcohol	21 (10.2)	1 (10.0)	
PSC/PBC/AIH	0/14/6 (9.7)	0/0/1 (10.0)	
NAFLD	1 (0.5)	0	
Others	25 (12.2)	1 (10.0)	
Ascites, *n* (%)			0.158
Mild	87 (42.4)	3 (30.0)	
Moderate	30 (14.6)	4 (40.0)	
Severe	19 (9.3)	0	
Indications for TIPS, *n* (%)			0.949
Recurrent variceal bleeding	181 (88.3)	9 (90.0)	
Refractory ascites	22 (10.7)	1 (10.0)	
Others	2 (1.0)	0	
Laboratory			
Hemoglobin (g/L)	85 (73–105)	70 (63–101)	0.112
White blood cell (×10^9^/L)	2.2 (2.1–3.3)	2.9 (2.0–3.3)	0.994
Total bilirubin (μmol/L)	23.1 (16.3–34.8)	24.4 (15.4–37.6)	0.477
Aspartate transaminase (U/L)	32 (23–41)	53 (40–60)	0.211
Alanine aminotransferase (U/L)	20 (13–30)	29 (20–34)	0.562
Albumin (g/L)	34.2 (30.5–38.6)	33.9 (23.5–38.0)	0.432
Creatinine (μmol/L)	70 (60–86)	66 (63–82)	0.331
Sodium (mmol/L)	138.9 (137.0–141.1)	140.2 (136.6–141.6)	0.591
INR	1.28 (1.20–1.39)	1.26 (1.08–1.37)	0.310
Fibrinogen (g/L)	1.55 (1.32–1.98)	1.81 (1.38–2.66)	0.294
Child-Pugh score	7 (6–9)	8 (6–10)	0.893
Child-Pugh class, *n* (%)			0.485
A (5–6)	50 (24.4)	2 (20.0)	
B (7–9)	125 (60.0)	5 (50.0)	
C (10–15)	32 (15.6)	3 (30.0)	
MELD score	11 (9–13)	11 (9–12)	0.604

### 3.2 The incidence of TIPS procedure-related hemorrhage in different PLT groups

**FIGURE 2:**
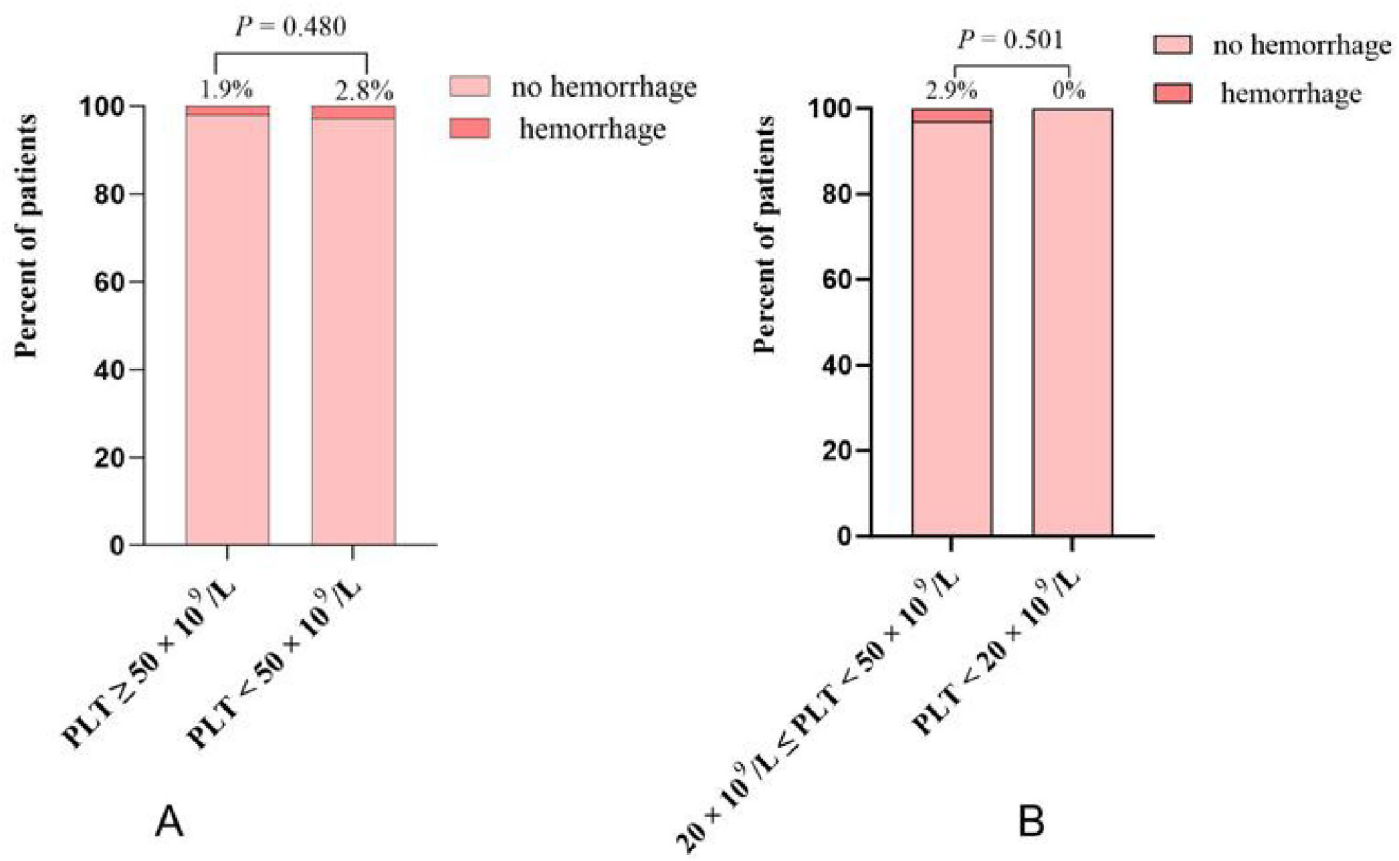
(A) The incidence of TIPS procedure-related hemorrhage in platelet (PLT) < 50 × 10^9^/L group and PLT ≥ 50 × 10^9^/L group; **(B)** the incidence of TIPS procedure-related hemorrhage in PLT < 20 × 10^9^/L group and PLT 20–50 × 10^9^/L group.

A total of 14 patients (2.2%) experienced TIPS procedure-related hemorrhage. Eight patients (1.9%) in the group with PLT counts ≥ 50 × 10^9^/L, and 6 patients (2.8%) in the group with PLT counts < 50 × 10^9^/L. There was no statistically significant difference between the two groups (CI: 0.23–1.97; *P* = 0.480) (Figure 2A). An episode of TIPS procedure-related hemorrhage was observed in 0 of the 10 patients in the PLT < 20 × 10^9^/L group and 6 of the 205 patients in the PLT 20–50 × 10^9^/L group (2.9%). Again, there was no statistically significant difference between these two groups (CI: 0–9.7; *P* = 0.501) (Figure 2B). Specific information of 14 patients with cirrhosis who experienced TIPS procedure-related hemorrhage is shown in Supplementary Table 1. Among these patients, 11 were women, and 7 were aged over 65 years. Bleeding occurred in 4 patients with CTP class A, 9 patients with CTP class B, 1 patients with CTP class C. Four patients who experienced bleeding had a MELD score ≥ 13, and none of the patients had a PLT count below 20 × 10^9^/L. Additionally, 12 patients experienced intraperitoneal bleeding, and 2 patients experienced hemobilia. Among them, 12 patients recovered gradually, 1 were transferred to the intermediate care unit (ICU) for further management, and 1 patient died.

### 3.3 Risk factors for TIPS procedure-related hemorrhage in decompensated cirrhosis

In the univariate analysis, increasing age (OR = 1.063, 95% CI: 1.015–1.112, *P* = 0.009) and CKD (OR = 6.271, 95% CI: 1.295–30.057, *P* = 0.023) were significantly associated with a higher risk of TIPS-related bleeding. Multivariate logistic regression analysis, adjusted for potential confounders (including age, CKD, PLT count, INR, Child score, MELD score, preoperative PPG, FIB and white blood cell), confirmed age (OR = 1.054, 95% CI: 1.006–1.105, *P* = 0.028) as the independent risk factor for TIPS-related bleeding (Table 3).

**TABLE 3:** Univariate and multivariate analyses.

Variable	Univariate analysis		Multivariate analysis	
	OR (95% CI)	*P*-value	OR (95% CI)	*P*-value
Age (years)	1.063 (1.015–1.112)	**0.009**	1.054 (1.006–1.105)	**0.028**
Chronic kidney disease	6.271 (1.295–30.357)	**0.023**	0.297 (0.057–1.551)	0.057
White blood cell (×10^9^/L)	1.161 (1.000–1.349)	**0.050**	1.125 (0.958–1.320)	0.151
Platelet count (×10^9^/L)	0.998 (0.988–1.007)	0.624		
Fibrinogen (g/L)	1.267 (0.969–1.656)	0.084		
INR	0.705 (0.036–13.994)	0.819		
Child score	1.044 (0.763–1.428)	0.790		
MELD score	1.043 (0.933–1.167)	0.459		
Preoperative PPG (mmHg)	1.062 (0.929–1.215)	0.379		

Values in bold indicate statistically significant differences (*P*≤0.05).

## 4 Discussion

These results from our retrospective study indicated that the incidence of TIPS procedure-related hemorrhage among cirrhotic patients is not directly associated with the PLT count, regardless of whether the PLT count is less than 50 × 10^9^/L or 20 × 10^9^/L. Advanced age was identified as an independent risk factor for TIPS procedure-related hemorrhage.

Given the lack of definitive evidence on the safety and efficacy of interventions to increase PLT counts in cirrhosis, an individualized approach is recommended for patients with severe thrombocytopenia prior to procedures. A study of 50 liver transplant candidates revealed that bleeding during invasive procedures occurred most frequently in patients with severe thrombocytopenia (PLT counts < 75 × 10^9^/L) (16). However, since the study involved patients undergoing various invasive procedures with different bleeding risks and included only 5 TIPS procedures, the findings may not be applicable to all cirrhotic patients who undergo TIPS. Bureau et al. (20) also reported that a PLT count above 75 × 10^9^/L is predictive of survival in patients following TIPS for refractory ascites. However, a study involving 1,100 therapeutic paracentesis in 628 patients, of which 598 had a PLT count less than 50 × 10^9^/L, showed no bleeding in any of the patients (21). In a study involving 150 patients with cirrhosis who underwent esophageal variceal ligation (EVL), thrombocytopenia was not identified as a predictive factor for EVL-related bleeding complications (22). He et al. (23) investigated the association between thrombocytopenia and the failure of endoscopic variceal treatment (EVT). They reported that thrombocytopenia may not significantly affect the efficacy of EVT in cirrhotic patients with acute variceal bleeding (AVB). Similarly, the main finding of our study was that thrombocytopenia may not be associated with TIPS procedure-related hemorrhage among cirrhotic patients. A major explanation for our finding should be that the rebalancing of hemorrhagic and coagulative processes in patients with cirrhosis. PLT function was often preserved in cirrhotic patients with thrombocytopenia and compensatory elevation of VWF has been observed in cirrhotic thrombocytopenia (2, 20, 24).

Systemic factors such as age, CKD, antiplatelet or anticoagulant use, and infection increase bleeding risk (25, 26). Similarly, our study revealed that advanced age is an independent risk factor for TIPS-related hemorrhage. Our study of 14 cirrhotic patients who experienced TIPS-related hemorrhage confirmed that 50% were over 65 years old. Advanced age is a recognized risk factor for fitness, attributable to an overall decline in physiological reserve (27). This includes reduced hepatic regenerative capacity, increased vascular fragility, and diminished cardiocirculatory compensation, which collectively elevate the risk of procedural complications like hemorrhage (28, 29). Age is also incorporated into the Freiburg index of post-TIPS survival (FIPS) to identify high-risk patients before TIPS placement (30).

As compared to previous studies, our study had several advantages in terms of study design. First, we exclusively enrolled a homogeneous cohort of cirrhotic patients undergoing TIPS to specifically investigate the association between preprocedural thrombocytopenia and procedure-related hemorrhage. Second, we stratified thrombocytopenia by severity, enabling an analysis of the relationship between different grades of low PLT counts and hemorrhagic risk. Third, our analysis mitigates confounding from variations in provider experience and technical approach, which could significantly influence patient outcomes.

Our study also had several limitations that should be acknowledged. First, the retrospective design inherently carries the potential for bias in sample selection. Secondly, the small sample size of the PLT count < 20 × 10^9^/L group means that we cannot entirely rule out the possibility that any observed differences between the two groups are a result of sample size limitations. In addition, within the group with PLT < 20 × 10^9^/L, no patients had a PLT < 10 × 10^9^/L, preventing us from making a recommendation on whether a PLT < 10 × 10^9^/L prior to TIPS increases the risk of TIPS-related hemorrhage. Third, given that the study is unicentric, the results should be interpreted with caution when applied to other centers, and further multicenter studies are needed to evaluate the predictive value of PLT counts for TIPS procedure-related hemorrhage. Fourth, given the retrospective nature of this study and the generally poorer liver function and coagulation synthesis in PLT < 50 × 10^9^/L group compared to PLT ≥ 50 × 10^9^/L group, baseline imbalances in INR, PT, FIB, Child-Pugh score, and MELD score were observed. Nevertheless, the study revealed no significant difference in TIPS-related bleeding rates between the two groups, despite the worse liver function and coagulation profile in PLT < 50 × 10^9^/L group. This finding is consistent with a previous study that did not identify these factors as risk factors for bleeding (15).

## 5 Conclusion

Our results indicate that the PLT count before TIPS placement is not associated with TIPS procedure-related hemorrhage. During preoperative assessment for TIPS, thrombocytopenia should not be considered an absolute contraindication. Advanced age is an independent risk factors for hemorrhage associated with TIPS.

## Data Availability

The original contributions presented in this study are included in this article/Supplementary material, further inquiries can be directed to the corresponding authors.
