# Supplementary material for: Association between preoperative platelet count and transjugular intrahepatic portosystemic shunt procedure-related hemorrhage in cirrhotic patients: a retrospective study
Source: Front Med (Lausanne). 2025 Nov 18;12:1683046. doi: 10.3389/fmed.2025.1683046 (PMC12669100; doi:10.3389/fmed.2025.1683046)
Supplement: Supplementary file 1 [file Table_1.docx]

| **Supplementary Table 1 Detailed Information of 14 Patients with Procedure-Related Hemorrhage** | | | | | | | | | | | | | | |
| --- | --- | --- | --- | --- | --- | --- | --- | --- | --- | --- | --- | --- | --- | --- |
| **Patient ID** | 1 | 2 | 3 | 4 | 5 | 6 | 7 | 8 | 9 | 10 | 11 | 12 | 13 | 14 |
| **Baseline Characteristics** | | | | | | | | | | | | | | |
| Sex | F | M | M | F | F | F | F | F | F | F | F | F | M | F |
| Age | 41 | 52 | 64 | 55 | 63 | 79 | 73 | 79 | 59 | 69 | 74 | 69 | 41 | 79 |
| MELD | 10 | 11 | 10 | 12 | 10 | 16 | 10 | 14 | 13 | 9 | 8 | 11 | 15 | 9 |
| Child-Pugh | A | B | B | B | B | B | B | A | B | B | A | B | C | A |
| Etiology | HBV | HBV | HBV | CC | AIH | CC | HBV | AIH | PBC | HBV | AIH | AIH | AC | AIH |
| Comordities^a^ | N | DM | N | N | HTN | CKD, DM | HTN | CKD, DM,  HTN | N | N | N | N | N | N |
| INR | 1.35 | 1.27 | 1.31 | 1.26 | 1.21 | 1.13 | 1.39 | 1.17 | 1.35 | 1.25 | 0.91 | 1.20 | 1.72 | 1.10 |
| FIB (g/L) | 1.86 | 1.89 | 1.52 | 0.99 | 1.70 | 6.04 | 2.12 | 5.32 | 1.42 | 1.20 | 3.95 | 3.43 | 1.09 | 1.60 |
| Pre-PLT (×10^9^/L) | 70 | 28 | 42 | 116 | 48 | 144 | 58 | 92 | 20 | 46 | 212 | 68 | 48 | 63 |
| Pre-HB (g/L) | 90 | 91 | 68 | 81 | 105 | 87 | 75 | 104 | 106 | 75 | 94 | 84 | 69 | 80 |
|  | | | | | | | | | | | | | | |
| **Detailed TIPS Parameters** | | | | | | | | | | | | | | |
| Indication | RVB | RVB | RVB | RVB | RVB | RVB | RVB | RA | RVB | RVB | RA | RA | RA | RVB |
| Anesthesia | LA | LA | LA | LA | GA | GA | GA | GA | GA | GA | GA | GA | GA | LA |
| Hepatic Vein  Approach | RHV | RHV | MHV | MHV | MHV | MHV | RHV | RHV | RHV | RHV | RHV | RHV | RHV | RHV |
| Number of | 1 | 1 | 1 | 1 | 1 | 1 | 1 | 1 | 1 | 1 | 1 | 1 | 1 | 1 |
| Punctures |  |  |  |  |  |  |  |  |  |  |  |  |  |  |
| **Patient ID** | **1** | **2** | **3** | **4** | **5** | **6** | **7** | **8** | **9** | **10** | **11** | **12** | **13** | **14** |
| Embolization of Varices | N | N | Y | Y | Y | Y | N | Y | Y | Y | Y | Y | Y | N |
| PPG (Pre/Post) | 20/6 | 22/8 | 18/8 | 22/10 | 16/6 | 29/11 | 20/6 | 21/8 | 23/8 | 21/1 | 19/5 | 22/8 | 20/6 | 25/10 |
| Stent Type | VC | VC | VC, BMS | VC, BMS | VC, BMS | VC | VC | VC | VC | VC | VC | VC | VC | VC |
| Stent Count | 1 | 1 | 2 | 2 | 2 | 1 | 2 | 1 | 1 | 1 | 1 | 1 | 1 | 1 |
| Stent Position | PV | PV | LPV | RPV | RPV | RPV | LPV | LPV | PV | RPV | RPV | RPV | RPV | LPV |
| Stent Diameter(mm) | 8 | 8 | Both 8 | Both 8 | Both 8 | 8 | Both 10 | 8 | 8 | 8 | 8 | 8 | 8 | 8 |
| Stent Length (mm) | 60 | 60 | VC:40  BMS: 80 | VC:60  BMS: 80 | VC:40  BMS:80 | 60 | VC1:60 VC2:40 | 70 | 70 | 60 | 50 | 60 | 80 | 80 |
|  | | | | | | | | | | | | | | |
| **Bleeding Outcomes** | | | | | | | | | | | | | | |
| Time | POD 0 | Intra-op | Intra-op | POD 1 | Intra-op | POD 1 | Intro-op | POD 2 | POD1 | POD 4 | POD2 | POD 7 | POD 7 | Intra-op |
| Type | IPH | IPH | IPH | IPH | IPH | IPH | IPH | IPH | IPH | IPH | IPH | Hemo-bilia | Hemo-bilia | IPH |
| Diagnosis^c^ | AP, CT/US | AP | AP | CT/US | AP, Angio | AP, Angio | Angio | AP | Angio | AP | AP | AP | CT/US | AP |
| Angiographic Localization |  |  |  |  | HA, SA, SMA | RHA | LPV |  | RHA |  |  |  |  |  |
| Clinical  Manifestations^b^ | 1,2,3 | 1 | 1 | 1,4,5 | 1 | 1 | 1,2 | 1 | 1 | 1 | 1 | 6,7 | 1 | 1 |
| Post-HB (g/L) | 63 | 37 | 21 | 74 | 57 | 76 | 71 | 94 | 84 | 73 | 86 | 79 | 58 | 40 |
| Outcome | CR | Death | CR | CR | CR | CR | CR | CR | CR | CR | CR | CR | ICU | CR |
|  |  |  |  |  |  |  |  |  |  |  |  |  |  |  |
| F: female; M: male; HBV: hepatitis B virus; CC: cryptogenic cirrhosis; AIH: autoimmune hepatitis; PBC: primary biliary cholangitis; AC: alcoholic cirrhosis; CM: comorbidity; CKD: chronic kidney disease; DM: diabetes mellitus; HTN: hypertension; RA: refractory ascites; RVB: recurrent variceal bleeding; GA: general anesthesia; LA: local anesthesia; RHV: right hepatic vein; MHV: middle hepatic vein; LHV: left hepatic vein; N: no/none; Y: yes; VC: Viatorr® covered stent; BMS: Bare-metal stent; PV: portal vein; LPV: left portal vein; RPV: right portal vein; POD: postoperative day; Intra-op: intraperative period; IPH: intraperitoneal hemorrhage; AP: abdominal paracentesis; CT: computed tomography; US: Ultrasound; Angio: angiography; HA: hepatic artery; SA: splenic artery; SMA: superior mesenteric artery; RHA: right hepatic artery; LPV: left portal vein; CR: clinical recover.  ^a^: Our analysis concentrated on three principal comorbidities: chronic kidney disease, diabetes mellitus, and hypertension.  ^b^: Clinical manifestations observed concurrent with hemorrhage detection included: 1: hypotension; 2: tachycardia; 3: cold/clammy skin; 4: fever; 5: ecchymosis; 6: abdominal pain; 7: melena.  ^c^: The diagnosis of hemorrhage type is primarily established through the following three modalities: abdominal paracentesis, CT/US, and angiography. AP: hemorrhagic ascites accompanied by hemodynamic instability (SBP <90 mmHg, HR >120 bpm) or HB drop ≥2 g/dL; CT/US: contrast-enhanced abdominal CT or ultrasonography demonstrating active extravasation or hematoma formation; Angio: direct visualization of contrast extravasation from hepatic vasculature. | | | | | | | | | | | | | | |
